# Simultaneous management of aortic and mitral regurgitation through one-stage transcatheter aortic valve replacement and transcatheter edge-to-edge repair: case report

**DOI:** 10.3389/fcvm.2024.1346022

**Published:** 2024-02-27

**Authors:** Hao Lin, Mei Zhu, Meng Lv, Zhengjun Wang

**Affiliations:** ^1^ Department of Cardiovascular Surgery, Shandong Provincial Hospital Affiliated to Shandong First Medical University, Jinan, Shandong, China; ^2^ Department of Ultrasound, Shandong Provincial Hospital Affiliated to Shandong First Medical University, Jinan, Shandong, China

**Keywords:** transcatheter edge-to-edge mitral valve repair, transcatheter aortic valve replacement, aortic insufficiency, mitral regurgitation, case report

## Abstract

This case report presents a 72-year-old male patient who presented with exertional dyspnea for over 10 years, which had progressively worsened over the past 4 months. Transthoracic echocardiography revealed severe aortic and mitral regurgitation, with a left ventricular ejection fraction of 37% and a left ventricular end-diastolic diameter of 64 mm. Despite receiving long-term optimal medical management, there was no improvement in symptoms or severity of valvular regurgitation. Given the relatively high surgical risk associated with double valve replacement in this elderly patient and his preference for minimally invasive procedures, a one-stage transapical aortic valve replacement and transcatheter mitral valve repair using the edge-to-edge technique were planned. The patient was discharged 8 days post-procedure without any complications. At 1-month follow-up, the patient's New York Heart Association (NYHA) functional class had improved to grade II.

## Introduction

A large, international prospective cohort study revealed that approximately 25% of patients with severe valvular heart disease had concomitant aortic valve and mitral valve disease (1). Concurrent moderate-to-severe mitral regurgitation (MR) occurs in approximately 25% of patients undergoing transcatheter aortic valve replacement (TAVR) and is an established risk factor for increased post-procedural mortality (2–4). Diagnosis can be challenging in these patients owing to the hemodynamic interaction between aortic regurgitation (AR) and MR. The increased end-systolic pressure from AR may overestimate MR severity, while the decreased forward ejection fraction from MR may underestimate AR severity, occasionally misclassifying severe AR as low-gradient AR. Acute severe AR exacerbates MR during left ventricular systole by increasing left ventricular end-diastolic pressure and impairing contractility, heightening the risk of MR. Acute regurgitation is more severe than chronic regurgitation and may escalate mild MR to severe levels. Aortic valve injury can delay mitral valve closure in systole, prolonging regurgitation.

The complexity of managing valvular heart disease is compounded when both the aortic and mitral valves are involved. TAVR has emerged as the primary treatment for high-risk aortic stenosis and regurgitation patients, supplanting surgical intervention as the standard of care (5). However, the optimal approach for patients with coexisting AR and MR is less clear, given the scarcity of data and the potential for diagnostic uncertainty. The hemodynamic interplay between severe AR and MR can exacerbate this uncertainty, leading to further diagnostic and therapeutic challenges (4).

Despite these challenges, recent evidence suggests that TAVR may lead to improvements in MR grade, although the prognostic implications of such improvements remain to be fully elucidated (6). This case report presents a patient who underwent TAVR with concurrent management of significant residual MR. Through this case, we aim to illuminate the intricacies involved in treating both conditions simultaneously and to contribute to the existing literature on the management of complex valvular heart disease. By exploring the potential benefits of a combined approach to AR and MR, this report adds valuable insights into the evolving landscape of interventional cardiology.

## Case presentation

**Figure 1:**
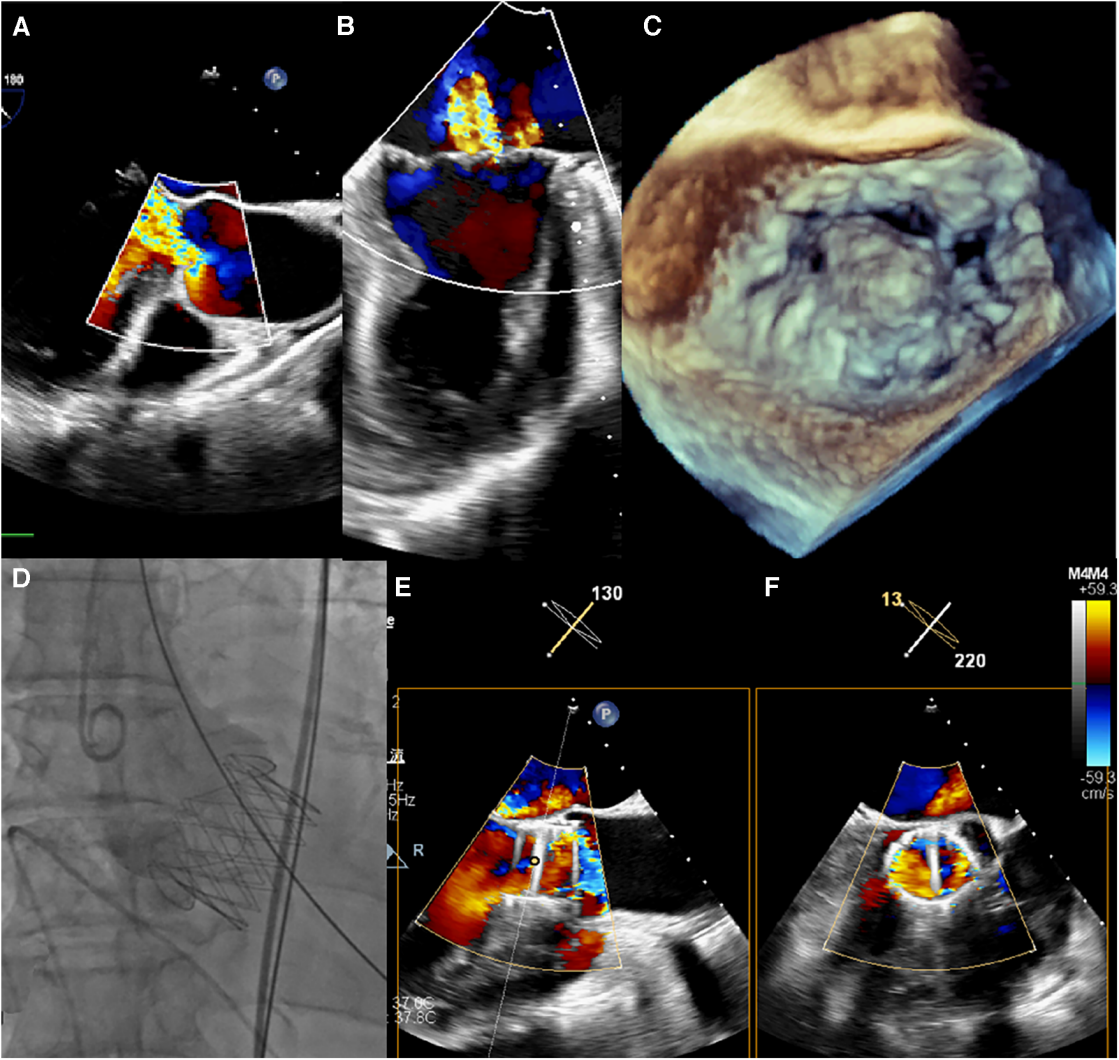
(**A–C**) intraoperative transoesophageal echocardiography confirmed severe aortic and MR. (**D**) Transapical TAVR was performed under fluoroscopic guidance using a 29 mm J-Valve (JieCheng Medical) as previously described, and aortic root angiography revealed minimal regurgitation. (**E,F**) After TAVR, the grade of MR remained severe.

This report presents a 72-year-old male patient who had experienced exertional dyspnea for over 10 years, worsening in the past 4 months. The patient is a 72-year-old male with a 10-year history of moderate AR and moderate MR, which were diagnosed 10 years ago. At that time, he was classified as New York Heart Association (NYHA) functional class II, and his left ventricular ejection fraction (LVEF) was approximately 50%. Due to patient preference, he declined surgical intervention and has been maintained on medical therapy. On follow-up echocardiography 2 years ago, there was slight worsening of his valvular regurgitation and his LVEF had declined to 45%. However, the patient opted to continue medical management. Over the past 4 months, he has developed worsening dyspnea on exertion and his functional status has deteriorated to NYHA class III-IV. On current evaluation, Transthoracic echocardiography revealed severe aortic and MR, with a left ventricular ejection fraction of 37% and a left ventricular end-diastolic diameter of 64 mm (Figures 1A–C).

He had 40-year history of smoking and long-term alcohol consumption. Optic nerve atrophy in the left eye was noted over 10 years ago. The patient reported a history of hematemesis 10 years prior, with unclear details, followed by intermittent melena since then without specific treatment. He had a definite medical history of hypertension for over 8 years. The patient suffered a cerebral infarction 1 year ago, with residual limb pain and numbness on the right side. Bilateral iliofemoral artery moderate to severe stenosis with varying degrees of arterial calcification was found on admission evaluation this time. The early medication regimen for heart failure treatment was as follows: metoprolol 25 mg orally twice daily, valsartan 80 mg orally once daily, spironolactone 20 mg orally once daily, furosemide 20 mg orally once daily. Over the past two years, the heart failure medication regimen was adjusted to: metoprolol 25 mg orally twice daily, sacubitril/valsartan 100 mg orally twice daily, spironolactone 20 mg orally once daily, furosemide 20 mg orally once daily, empagliflozin 10 mg orally once daily.

Given his high surgical risk, further assessment and decision-making will involve a multidisciplinary heart team including cardiologists, cardiac surgeons, anesthesiologists and other specialists. This group will evaluate his case details together, including the recent echocardiographic findings, and the cardiac surgeons will determine the optimal treatment plan. Given the relatively high risk of conventional open heart double valve surgery in this elderly individual and the patient's preference for minimally invasive procedures, a one-stage transapical aortic valve replacement (TAVR) and transcatheter edge-to-edge repair (TEER) were planned. The physician hoped that this one-stage TAVR combined with TEER surgery could maximize the benefits by replacing the aortic valve and repairing the mitral valve simultaneously, while minimizing surgical trauma and improving therapeutic outcomes. It was expected to markedly improve the patient's symptoms and prolong his survival.

The preoperative transthoracic echocardiography findings on admission showed: aortic valve prolapse with severe regurgitation, mitral valve prolapse with severe regurgitation, and pulmonary hypertension. The mitral valve ring was dilated to approximately 4.74 cm in diameter. Valve leaflet thickening was observed with echogenicity. Partial chordae tendineae were thinning in the valvular recess. P2 region and partial P1 region showed sac-like protrusion into the left atrium. The anterior leaflet measured approximately 2.36 cm in length and the posterior leaflet measured approximately 2.06 cm in length. The aortic valve exhibited a trileaflet structure. Valve leaflet thickening with echogenicity was observed. The right coronary cusp was slightly prolapsing downward in diastole. The aortic valve ring was measured at approximately 2.62 cm in diameter. The sinus of Valsalva was approximately 3.84 cm in internal diameter and the sinotubular junction was approximately 3.34 cm in internal diameter. Minor anechoic areas, suggestive of pericardial effusion, were detected in the pericardial sac. The inferior vena cava measured approximately 1.55 cm in internal diameter with approximately 50% collapse on respiration. The left ventricular outflow tract measured approximately 2.50 cm in internal diameter. Color flow Doppler imaging revealed severe regurgitant signals the aortic and mitral valves. The MR formed two eccentric jets, one arising from the prolapsing leaflet and the other located at the junction of the anterior and posterior mitral leaflets. Continuous wave Doppler measured the maximum aortic valve regurgitant velocity (Vmax) at 138 cm/s and the maximum pressure gradient (PGmax) at 8 mmHg. Severe MR signals were detected arising from the P2 region and partial P1 region with a vena contracta of approximately 0.7 cm. Mild tricuspid regurgitation signals were detected with a Vmax of 316 cm/s and PGmax of 40 mmHg, estimating the pulmonary artery systolic pressure at approximately 45 mmHg (Table 1).

**Table 1:** Clinical and echocardiographic baseline characteristics.

			Preoperative	1-month postoperative
Age (year)	72	Ascending aorta diameter (mm)	39.0	40.1
Past or current tobacco use	No	Aortic valve annulus diameter (mm)	26.2	–
Hypertension	Yes	Sinus of Valsalva diameter (mm)	38.4	–
Diabetes	No	Sinotubular junction diameter (mm)	33.4	–
Prior stroke	Yes	Left ventricular outflow tract diameter (mm)	25.0	–
Chronic obstructive pulmonary disease	No	Left atrial diameter (mm)	50.9	41.0
Prior myocardial infarction	No	Left ventricular diameter (mm)	64.0	60.4
Atrial fibrillation or flutter	No	Aortic valve peak velocity (mm/s)	138.0	171.0
Renal failure requiring dialysis	No	Maximum aortic valve pressure gradient (mm)	–	12.0
Peripheral arterial disease	Yes	Maximum mitral valve pressure gradient (mm)	–	5.0
EuroSCOR	8	Mitral valve annulus diameter (mm)	47.4	33.0
EuroSCORII	4.64	Anterior mitral leaflet length (mm)	23.6	–
		Posterior mitral leaflet length (mm)	20.6	–
		Mitral valve effective orifice area (cm^2^)	–	3.6
		Mitral valve regurgitant jet width (mm)	7.0	–
		Left ventricular ejection fraction (%)	37.0	431.0
		Estimated pulmonary artery systolic pressure(mmHg)	45.0	37.0

**Figure 2:**
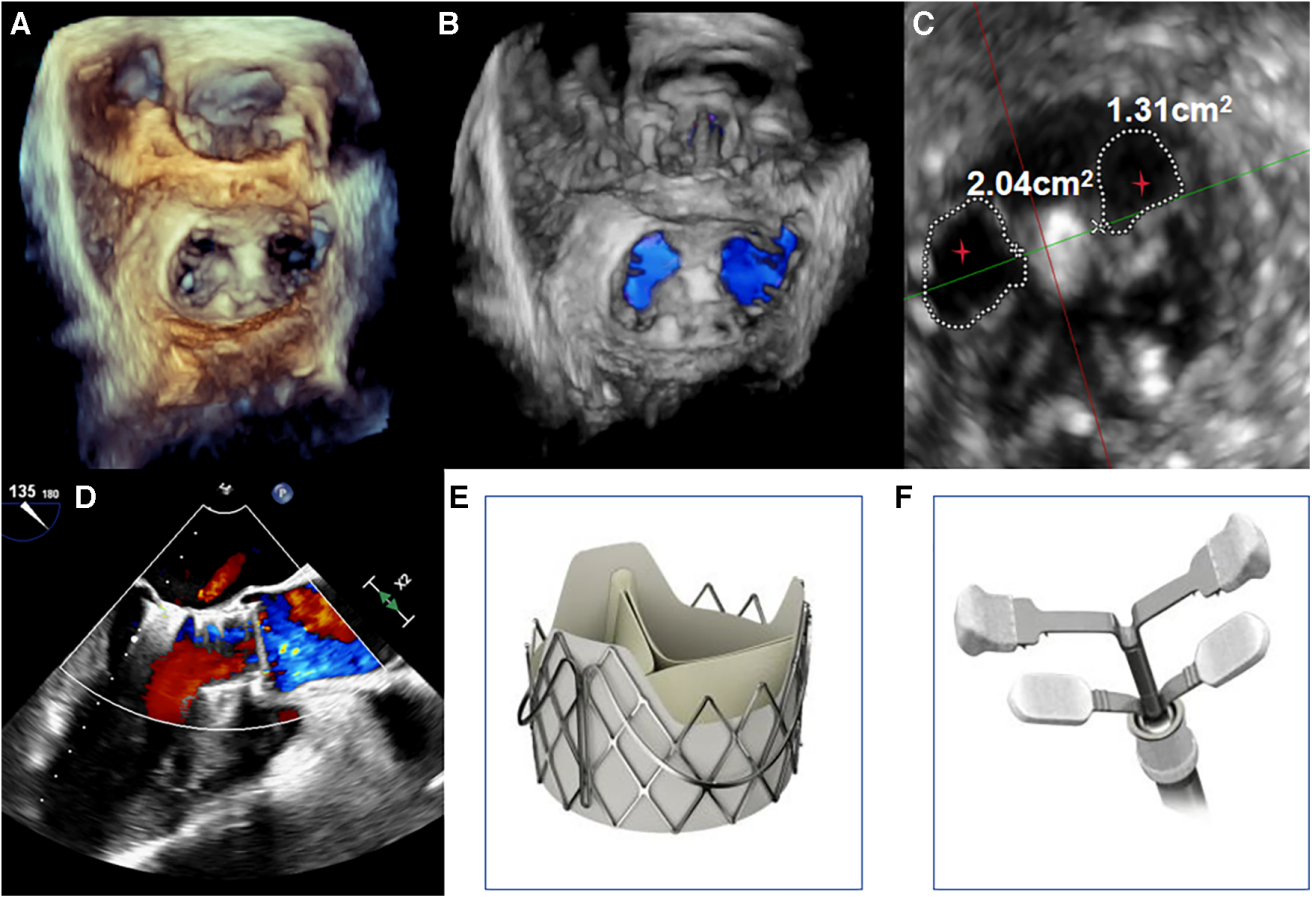
(**A,B**) under transoesophageal echocardiography guidance, one clip was implanted between the A2 and P2 leaflets. Transoesophageal echocardiography with 3D reconstruction demonstrated appropriate morphologies of the mitral and aortic valves post-TAVR and transcatheter edge-to-edge repair. (**C**) The mitral valve dual orifice areas measured by transoesophageal echocardiography were 2.04 cm^2^ and 1.31 cm^2^, respectively. The red stars in Figure depict the dual orifice areas of the mitral valve. (**D**) Transoesophageal echocardiography showed mild paravalvular regurgitation of the aortic valve after TAVI and TEER, with resolution of MR. The maximum transvalvular pressure gradient across the mitral valve was 3 mmHg, with a mean pressure gradient of 1 mmHg. (**E,F**) The transcatheter heart valves used were the J-Valve (JieCheng Medical, China) for the aortic position and the MVC-IIIf mitral valve clip (Shanghai Hanyu Medical, China) for the edge-to-edge repair.

The procedure was performed under general anesthesia. Under TEE guidance, a puncture site was located at the cardiac apex, and a hexagonal purse-string suture was placed around this point. A 6F sheath was inserted at the center of the purse-string suture using a J-wire under fluoroscopic guidance, and a JR4 catheter was advanced into the descending aorta. After exchanging for an Amplatz stiff wire, a 22F dilator was used to dilate the puncture site at the apex. Subsequently, under the guidance of the Amplatz wire, a 29# J-Valve (JieCheng Medical) was implanted at the level of the aortic valve annulus. Aortic root angiography revealed minimal regurgitation around the aortic valve, and the delivery sheath was carefully removed under fluoroscopy (Figure 1D, Figure 2E). After undergoing TAVR surgery, the level of MR remained severe (Figures 1E,F). A 16F sheath was then inserted through the original puncture site, and wire manipulation confirmed proper alignment towards the mitral valve. The Shanghai Hanyu Medical mitral valve delivery system DS-16F-165 was used to deliver the transcatheter mitral valve (model: KBQ-14) to the left atrium, and the delivery system was then withdrawn after the valve was positioned. The MVC-IIIf mitral valveclamp (Shanghai Hanyu Medical) was loaded onto the delivery system and advanced to the left atrium, where it was fully opened. Under transesophageal echocardiography guidance, the clip arms were positioned perpendicular to the mitral valve opening line, capturing the anterior and posterior leaflets of the mitral valve in the P2 and A2 regions. The clip was then partially closed in the left ventricle, securing the mitral valve leaflets. Two- and three-dimensional echocardiography confirmed the proper position of the mitral valve clip, with resolution of MR and optimal clip closure, resulting in a maximum transvalvular gradient of 3mmHg and a PGmean of 1 mmHg (Figure 2A,B). The dual orifice areas of the mitral valve, as measured by transesophageal echocardiography, were 2.04 cm^2^ and 1.31 cm^2^ respectively (Figure 2D). The clip was released, the delivery system was successfully removed, the purse-string suture was tied, and the chest was closed. The MitraClip system was positioned and secured with excellent performance and sealing integrity. The mitral valve clip was well-positioned and securely fixed (Figure 2D,F). The post-operative course was uneventful and without complication. The patient was discharged on post-operative day seven. After 1 months of follow-up, the patient's NYHA class improved to grade 2.

## Discussion

This case review focuses on the importance of transcatheter valve therapies, particularly TAVR and edge-to-edge repair (TEER), for treating complex valve diseases. These minimally invasive interventions provide an alternative for high-risk elderly patients who are not suitable candidates for open-heart surgery. Specifically, TEER has been shown to be an effective approach for treating severe MR (MR), similar to the Alfieri edge-to-edge repair technique used in surgical procedures.

Furthermore, while TAVR has been shown to improve MR grading, the long-term implications of this improvement on prognosis remain unclear. This case involves TAVR treatment in a patient with significant residual MR, indicating potential benefits for patients with concomitant AR and MR. Additionally, a detailed analysis is conducted on the hemodynamic interaction between AR and MR and its influence on diagnostic and treatment decisions. The presence of AR may lead to an overestimation of the severity of MR, while the reduction in forward ejection fraction caused by MR may lead to an overestimation of AR. Doldi et al. (7) conducted a study to evaluate the impact of MRetiology on the prognosis of patients undergoing transcatheter aortic valve implantation (TAVI). The study included 631 TAVI patients, categorized based on MR etiology into acute functional MR related to atrial functional MR (aFMR), ventricular functional MR related to left ventricular remodeling (vFMR), and primary MR (PMR) groups. The results demonstrated that the aFMR group exhibited the highest MR improvement rate (80.2%), which was significantly higher than the vFMR group (69.4%) and PMR group (40.8%) (both *p* < 0.05). The 3-year survival rate was similar across all groups. However, persistent MR was associated with an increased risk of mortality, primarily driven by the PMR subgroup. All groups exhibited varying degrees of improvement in cardiac function. The study underscores MR etiology as a crucial factor influencing post-TAVI prognosis, with PMR showing the poorest prognosis. Considering MR etiology facilitates risk assessment and decision-making in TAVI patients. In light of this literature, our patient's mitral valve pathology in the case report should be classified as PMR. Through a one-stage TAVR combined with TEER procedure, significant improvement in the symptoms and severity of MR can be expected. This is especially notable if the patient's MR etiology is associated with aFMR. The MR in this case is primary in nature, due to degenerative changes of the mitral valve leaflets. We do not believe that treating the aortic valve disease alone could fully resolve this primary MR, since aortic valve replacement does not correct the intrinsic abnormalities of the mitral valve itself. It is worth emphasizing the unique design features of the J-Valve system that make it well-suited for TAVR in our 72-year-old patient with severe AR. The J-Valve system is designed to offer positioning flexibility and anatomical compatibility, which may be beneficial in certain clinical scenarios. However, it is important to compare its performance and outcomes directly with other well-established devices such as the Sapien and MitraClip systems through further research. The J-Valve system features a movable clasper mechanism designed for precise adjustments post-deployment, aiming for accurate alignment with the aortic annulus (8). This design aspect aims to minimize the risk of coronary artery obstruction, a concern in patients with closely adjacent coronary ostia.Additionally, the clasper of the J-Valve system forms a “lock and key” relationship with the aortic annulus through its straight portion that aligns with the junction line of the native valve, aiding accurate anatomical positioning which is crucial to avoid complications such as paravalvular leak and valve malposition (9). The J-Valve system is a novel TAVR (TAVR) system (10). reported the 2-year follow-up results of 111 patients who underwent TAVR with the J-Valve. The study showed a 2-year survival rate of 92.5% and no cases of valve failure occurred. Additionally, Li et al. (11) conducted long-term follow-up of 43 patients, confirming the durability of the J-Valve system with a 5-year survival rate of 88.4%.

Based on our experience, the transapical implantation technique of the J-Valve system is relatively straightforward, requiring accurate measurement of the aortic annulus diameter and appropriate oversizing of the transcatheter heart valve (THV). In the two-stage implantation process, attention to detail is essential to ensure that the clasper does not trap the edge of the prosthesis, as this could lead to severe deformation, paravalvular leak, or valve malposition. A thorough check of the clasper configuration before deploying the prosthesis is recommended, and any involvement of the clasper should be addressed by adjusting the prosthesis upward, realigning the delivery system, and redeploying as necessary to rectify the issue.

A previous case report demonstrated the feasibility and efficacy of a double valve-in-valve procedure for a failed mitral and aortic bioprosthetic valve using a self-expanding valve via a transapical approach, where a J-Valve was utilized (12). Due to its large ventricular outflow tract opening and short stent frame design, the J-Valve was well-suited for this patient's anatomy and helped minimize the risk of left ventricular outflow tract obstruction. An additional benefit of this valve design is the potential for coronary preservation through maintaining adequate distance between the replacement valve leaflets and the coronary ostia, thus reducing the risk of coronary occlusion. While the J-Valve system may present a cost-effective option, it is essential to consider the overall value, including clinical outcomes and patient safety, in comparison to other established devices like the Sapien 3 valve. Its design characteristics may offer clinical advantages for patients requiring valve-in-valve procedures or valve replacement in complex anatomical situations, though further research is still needed.

The J-Valve system offers several advantages for TAVR procedures, particularly in complex anatomical cases. As demonstrated in our case, the valve's innovative design and deployment flexibility allow for customized sizing and positioning to optimize clinical outcomes (9). The system's movable clasper mechanism facilitates precise adjustments to ensure accurate alignment with the aortic annulus (8). This tailored approach is well-suited for treating AR, even in situations with anatomical challenges. It should be noted that due to severe calcification and stenosis of peripheral arteries, especially the iliofemoral arteries, in this patient, we did not choose a transfemoral approach for TAVR using systems like the JenaValve. Such systems rely on adequate diameter and compliance of the iliofemoral vessels as the access route. The iliofemoral arterial pathology in this case contraindicates the transfemoral approach.

The ValveClamp system (HanYu Medical Technology, Shanghai, China), a new transcatheter edge-to-edge mitral valve repair device, is introduced as an alternative to existing technologies (13). ValveClamp is delivered via a transapical approach and can be easily aligned coaxially with the mitral valve by selecting an appropriate apical puncture site (guided by echocardiography). This approach is both short and direct, and the adjustment of the clamping position is straightforward. It features arms that move parallel to each other for leaflet grasping, differing from the V-shaped deployment seen in the MitraClip. This design aims to offer a broader capture range, potentially simplifying certain repair procedures. This allows the arms to fully open, resulting in a much larger capture range compared to the MitraClip. These factors may make the ValveClamp procedure easier than the traditional transseptal edge-to-edge mitral valve repair surgery. In cases that were considered challenging and excluded from MitraClip clinical trials, such as leaflet cleft width ≥10 mm or cleft length ≥15 mm, ValveClamp has not excluded these patients due to its larger capture range. The edge-to-edge mitral valve repair device is a novel interventional therapy for MR. Pan et al. (13) first applied this device in humans, with preliminary results demonstrating its safety and efficacy. Subsequently, studies by Ge et al. (14) involving real-time monitoring and step-by-step guidance as well as long-term follow-up also validated the potential of this system in simplifying the surgical process and reducing MR. Ge et al. (14) further confirmed that this device can effectively improve the three-dimensional geometry of the mitral valve. In summary, data from these validation trials provide strong evidence to support the clinical application of the J-Valve system and edge-to-edge mitral valve repair device.

The management of patients diagnosed with concurrent significant MR and AR poses a significant clinical challenge due to the complexity of the dual valve disease. While surgical interventions have traditionally been the mainstay of treatment, the emergence of transcatheter therapies has begun to offer promising alternatives. Current guidelines lack robust evidence to guide the management of combined AR and MR. In clinical practice, AR and MR may present as both severe, severe AR and moderate MR, or severe MR and moderate AR. Mild lesions only require monitoring (15). In cases of severe AR and MR, treatment of both valves is warranted. Mitral repair/replacement is recommended as Class I for primary MR with AR replacement (Level B evidence), but as Class IIa for secondary MR (Level C). AR replacement is recommended as Class I with MR surgery (Level C). In cases of severe AR and moderate MR, severe AR is treated according to guidelines. Concomitant surgery for moderate secondary MR lacks evidence for survival benefit (Class IIb, Level C). The likelihood of MR improvement after AR replacement also raises questions about additional surgery. Valve repair is preferred over replacement for moderate primary MR (Class IIa, Level C). In cases of severe MR and moderate AR, severe MR is treated according to guidelines. Concomitant AR surgery is reasonable to prevent progressive worsening (Class IIa, Level C). Occasionally, two moderate lesions can still have significant repercussions. No studies guide the management of this scenario. Nevertheless, a thorough assessment is warranted, and valve surgery may be considered if the global hemodynamic impact is significant, after accounting for confounders such as lung disease or ventricular dysfunction. Percutaneous mitral valve repair has demonstrated safety and efficacy. Recent evidence indicates that TAVR may be feasible for high surgical risk native valve AR, although it is not yet standard practice. However, fully transcatheter management of both conditions is rare. A review of 37 studies involving 60 high-risk or inoperable patients undergoing combined transcatheter aortic and mitral intervention found that only 4 (7%) had both aortic and MR, with just one undergoing a transcatheter approach on native valves (15). There is limited data guiding the management of combined aortic and MR. Left ventricular dysfunction is common at presentation and postoperatively, suggesting that surgical delays, especially with symptoms or ventricular dysfunction, should be avoided. As with other combined valve diseases, multidisciplinary decisions optimize outcomes. While current transcatheter roles are limited, advances are likely to change paradigms.

## Conclusion

This case report demonstrates the feasibility and effectiveness of performing TAVR and TEER concurrently in a selected patient with severe AR and MR.

## Data Availability

The raw data supporting the conclusions of this article will be made available by the authors, without undue reservation.
